# Scanner-Measured Hand Hygiene Performance After Automated Visual Feedback-Supported Training: A Multicenter Quality Improvement Project in Italian Hospitals

**DOI:** 10.3390/nursrep16080275

**Published:** 2026-08-05

**Authors:** Yari Longobucco, Giulia Adriano, Giuseppe Pedrazzi, Giulia Di Mari, Margherita Boschetto, Mariagrazia Paoletti, Maria Carmela Santarsiero, Federica Scola, Maria Mongardi

**Affiliations:** 1 Department of Health Sciences, University of Florence, 50134 Florence, Italy; 2 ANIPIO, National Association of Nurses for the Prevention of Hospital Infections, 40122 Bologna, Italy; giulia.adriano@galliera.it (G.A.); maria.santarsiero@galliera.it (M.C.S.);; 3 Infectious Disease Unit, Galliera Hospital, 16128 Genoa, Italy; 4 Unit of Neuroscience & Interdepartmental Center of Robust Statistics, Department of Medicine and Surgery, University of Parma, 43121 Parma, Italy; 5 Epidemiology and Infectious Disease Unit, University Hospital of Padua, 35128 Padua, Italy; margherita.boschetto@gmail.com; 6 Infectious Disease Unit, Policlinico San Martino Hospital, 16132 Genoa, Italy; 7 Department of Medicine and Surgery, University of Parma, 43121 Parma, Italy

**Keywords:** hand hygiene, automated feedback, visual feedback, infection control, nursing education, quality improvement, healthcare-associated infections

## Abstract

**Background:** Healthcare-associated infections (HAIs) remain a major patient safety issue, and hand hygiene (HH) is a key preventive measure. Technologies providing immediate visual feedback may support HH training by allowing objective assessment of hand-rubbing technique. **Methods:** A multicenter quality improvement project was conducted in three hospitals in northern Italy to assess scanner-measured HH performance over time after a standardized HH training program supported by an automated visual feedback scanner, the Semmelweis Hand Hygiene System^®^. Nurses and nurse aides performed scanner assessments before training (T0) and up to 6–9 months after training (T1–T4). Data included demographic variables, baseline HH knowledge, ring detection, and scanner pass/fail outcomes. Longitudinal changes were analyzed using adjusted generalized estimating equation (GEE) logistic regression models. **Results:** A total of 292 healthcare professionals participated in the project (69.2% nurses; mean age 42 years; 81.1% female). At baseline, 44.2% failed the scanner assessment, with dorsum areas being the most frequently insufficiently covered, and 36.0% wore rings. Across 2422 scanner assessments, pass rates improved over time. In adjusted GEE models, each additional scheduled month from baseline was associated with higher odds of passing the scanner assessment (OR = 1.18, 95% CI 1.13–1.24, *p* < 0.001). This association remained significant in the first-scan-only sensitivity analysis (OR = 1.16, 95% CI 1.11–1.21, *p* < 0.001). The association was also confirmed in a completers-only sensitivity analysis. Ring detection was associated with lower odds of passing, whereas baseline HH knowledge was not independently associated with scanner performance. **Conclusions:** Scanner-based immediate feedback was associated with improved scanner-measured HH technique over time. However, the quality improvement design, absence of a control group, use of the scanner as both feedback tool and outcome measure, and declining retention across follow-up assessments limit causal interpretation. Scanner-supported feedback may represent a useful adjunct to traditional HH education, but further controlled studies are needed to assess sustainability and real-world HH compliance.

## 1. Introduction

Healthcare-associated Infections (HAIs) remain a major challenge in healthcare delivery [1]. HAIs are infections that occur 48 h or more after hospital admission, or after discharge in specific circumstances, such as surgical site infections occurring within the relevant surveillance period [2]. Common types of HAIs include catheter-associated urinary tract infections, central line-associated bloodstream infections, surgical site infections, ventilator-associated pneumonia, and *Clostridioides difficile* infections [2]. These infections contribute to increased morbidity, prolonged hospital stays, higher healthcare costs, and avoidable mortality [1,2]. In Italy, HAIs also represent a relevant patient safety issue, with reported incidence varying across settings and patient populations [1,3,4]. Hand hygiene (HH), including the use of alcohol-based hand rub, remains one of the most important measures for preventing HAIs [5]. The World Health Organization (WHO) has promoted the “My Five Moments for Hand Hygiene” framework to guide HH practice in clinical care [6]. However, healthcare workers’ HH compliance remains suboptimal in many settings, frequently falling below recommended standards [7]. Importantly, gaps in HH practice are not explained by knowledge alone. Although education can improve awareness and adherence, contextual factors such as workload, time pressure, resource constraints, and local safety culture may hinder consistent practice [7]. Multimodal approaches, including education, feedback, reminders, system change, and safety culture, are recommended to improve HH behavior and patient safety [7]. Within this framework, technologies providing immediate visual feedback may offer an additional educational strategy by making HH technique more observable and actionable. Interactive scanners and ultraviolet- or fluorescence-based systems can objectively identify areas of the hands that are insufficiently covered during hand rubbing, allowing healthcare workers to immediately recognize and correct technique gaps [8]. These tools do not measure real-world compliance with the WHO Five Moments, but rather assess the technical quality of hand rubbing under test conditions. In recent years, information technology and automated hand hygiene monitoring systems have increasingly been explored as supports for HH education, monitoring, reminders, and feedback, although evidence remains heterogeneous and implementation in routine care is complex [8,9,10]. However, evidence on scanner-supported HH training in Italian multicenter quality improvement settings remains limited. Therefore, the aim of this project was to assess scanner-measured HH performance over time after a standardized HH training program supported by an automated visual feedback scanner among nurses and nurse aides in three Italian hospital centers.

## 2. Materials and Methods

### 2.1. Study Design and Sample

The study was designed as a multicenter quality improvement (QI) project conducted in three hospital centers in northern Italy. Participating sites were selected by convenience because the Semmelweis Hand Hygiene Scanner^®^ (HandInScan Zrt., Debrecen, Hungary) was already available locally for the implementation of structured HH training activities in all three centers. The project was conducted as part of a QI initiative promoted by ANIPIO, the Italian National Scientific Society of Nurses Specialists in Infectious Risk. Within each participating center, the clinical areas involved in the project were identified by local IPC nurses according to local QI priorities, organizational feasibility, and scanner availability. The HH training course was offered to nurses and nurse aides working in these selected clinical areas through the nursing coordinators. Participation was voluntary and occurred within routine professional training and QI activities. No separate sampling frame for all potentially eligible healthcare workers was created, and the total number of eligible staff members in the participating wards was not available to the research team; therefore, a recruitment rate could not be calculated.

The project involved nurses and nurse aides working in three clinical areas with different intensities of care: internal medicine/geriatrics, surgery, and intensive care. These professional groups were included because they represented the target population of the local HH training programs. Because the initiative was embedded in routine professional training and QI activities, no formal a priori sample size calculation was performed. All staff members who participated in the training and completed the baseline scanner assessment were included.

Participation was voluntary, and participants were informed about the purpose and procedures of the project. In accordance with the QI nature of the initiative, formal written consent was not required; voluntary completion of the training activities and related assessments was considered agreement to participate. No directly identifiable personal data were collected. To allow longitudinal tracking while preserving non-identifiability, each participant was assigned a unique study code during the initial session. This code was used for all subsequent assessments without collecting names, contact details, or other direct personal identifiers. According to QI reporting principles [11], the three centers were anonymized in Center #1, Center #2 and Center #3.

### 2.2. Measurements

#### 2.2.1. Semmelweis Hand Hygiene System^®^

The Semmelweis Hand Hygiene Scanner^®^ (HandInScan Zrt., Debrecen, Hungary) is a manufacturer-described AI-driven, automated image-based scanner that provides immediate visual feedback on hand-rubbing technique. In operational terms, the automated image-based analysis identifies hand areas covered and not covered by fluorescein-based gel under UV-A illumination and classifies the assessment according to the percentage of covered hand surface. During the assessment, participants apply a fluorescein-based alcohol solution simulating alcohol-based hand rub. The scanner uses a harmless ultraviolet A (UV-A) light source to identify covered and uncovered hand areas and provides an automated evaluation of HH technique. The system generates an overall pass/fail result and area-specific feedback for the left and right palm and the left and right dorsum. A test is classified as passed when at least 95% of the total hand area is covered by the gel. The system can also detect the presence of rings. The ultraviolet-dye-based approach underlying this type of assessment has been microbiologically validated in previous studies assessing hand hygiene technique [12,13,14]. The scanners were subject to routine periodic maintenance according to local procedures. No study-specific calibration was performed by the research team, and technical maintenance records were not available for analysis. Before each training or follow-up session, local IPC nurses performed functional checks to verify correct device start-up, software operation, UV-A illumination, and availability of the fluorescein-based solution. The same type of fluorescein-based solution was used across assessments, and scanner use followed the standardized procedure shown during training, consistent with the manufacturer’s operating instructions.

#### 2.2.2. Questionnaires

The Italian version of the “WHO Hand Hygiene Knowledge Questionnaire for Health Care Workers” was used [15]. The questionnaire is a WHO training and monitoring tool based on the WHO hand hygiene guidelines and includes 26 numbered questions, several of which comprise multiple scoreable sub-items. Italian adaptations of this questionnaire have been used in previous observational studies among healthcare workers [16,17]; however, to our knowledge, no complete psychometric validation study of the Italian version has been published. Therefore, in the present project, internal consistency of the 46 dichotomously scored elements was assessed using KR-20. Each correct response was assigned 1 point and each incorrect or missing response 0 points, yielding a total knowledge score ranging from 0 to 46, with higher scores indicating greater HH knowledge.

Additional information was collected on gender, age, profession (nurse or nurse aides), work setting (internal medicine/geriatrics, surgery, or intensive care unit), and previous participation in HH training courses.

### 2.3. Study Procedures

The training consisted of a standardized frontal lecture delivered across the three centers using the same slides, materials, contents, and duration. The training program was developed by the research group and the Scientific Committee of the Italian Society of Nurses involved in Infection Prevention and Control (IPC) of ANIPIO. In each center, the training was delivered by local IPC nurses who were also ANIPIO members. The main contents covered were: (i) definition and impact of HAIs; (ii) main modes of germ transmission; (iii) HH and HAI prevention; and (iv) how, when, and why to perform HH.

Before the lecture, participants first completed a baseline scanner assessment (T0), followed by administration of the questionnaire, both conducted within the same session. The training session was delivered immediately afterward. No prior practice with the scanner was provided before the baseline assessment, which was intended to reflect participants’ existing hand-rubbing technique under test conditions. At the end of the theoretical training, participants were allowed to practice with the scanner and receive immediate visual feedback to refine their technique.

During the training, based on WHO HH recommendations, participants were informed about the importance of removing hand and wrist jewelry. However, they were not required to remove rings before scanner assessments, in order to reflect routine clinical practice in a real-world QI setting. Ring presence was recorded at each scanner assessment and included in the analysis.

After the training, the scanner was made available to the participating departments on a rotating basis to conduct follow-up assessments. All centers performed at least three follow-up periods, at approximately 2 months (T1), 4 months (T2), and 6 months (T3) after training. At each time-point, participants were asked to perform up to three repeated scanner assessments, labelled for example as T1.1, T1.2, and T1.3. Two centers performed an additional follow-up assessment at approximately 9 months (T4). The number of assessments performed varied across centers and time-points because of organizational factors and differences in local training plans, as expected in QI projects [11]. No formal assessment of institutional IPC maturity, such as IPCAF, was performed before implementation.

### 2.4. Data Analysis

Data were described using absolute and relative frequencies or means and standard deviations, depending on the type of variable. Given the presence of missing data, the number of observations is reported for each variable. Baseline differences between groups were assessed using chi-square tests, ANOVA, or equivalent non-parametric tests, as appropriate.

Longitudinal changes in scanner performance were analyzed using generalized estimating equation (GEE) logistic regression models with a binomial distribution and logit link, robust standard errors clustered by participant ID, and an exchangeable working correlation structure. The dependent variable was scanner test success (pass/fail) for each assessment. Time was modeled both as a continuous variable using scheduled months from baseline (0, 2, 4, 6, and 9 months; repeated assessments within the same follow-up period were assigned the same nominal month value) and categorically by follow-up period. Center was included as a fixed effect to account for inter-center heterogeneity. Because participants were nested within three hospital centers, potential hospital-level heterogeneity was addressed by including center as a fixed effect in all adjusted models. Robust standard errors were clustered at the participant level to account for repeated assessments within individuals. Cluster-robust standard errors or random effects at the hospital level were not used because only three centers were included, which would provide an insufficient number of clusters for reliable estimation. Adjusted models included profession, gender, age, previous HH training, baseline HH knowledge score, and ring detection. Because some baseline covariates were missing, adjusted models were conducted as complete-case analyses. Missing data were not imputed. For descriptive analyses, available case denominators are reported for each variable. Baseline scanner outcome, profession, work setting, and center were complete for all 292 participants. Missingness affected gender, age, previous HH training, and baseline HH knowledge score. Missing follow-up scanner assessments were treated as not observed and were addressed descriptively through retention analyses and analytically through sensitivity analyses.

To assess the potential influence of within-session repeated practice, a sensitivity analysis was restricted to the first scanner assessment of each follow-up period. Retention analyses were performed to assess whether loss to follow-up was potentially selective, comparing baseline characteristics between participants who did and did not complete follow-up assessments. Because standard unweighted GEE models assume non-informative missingness of outcome data conditional on the model structure, and because retention analyses suggested selective loss to follow-up, an additional completers-only sensitivity analysis was conducted among participants who completed the final common follow-up assessment at T3.3. Because ring detection could plausibly be influenced by the training itself, an additional sensitivity model was fitted without ring detection as a covariate. Statistical analyses were performed using Stata version 13.

## 3. Results

The characteristics of the sample are reported in Table 1. A total of 292 healthcare professionals participated in the project. Most participants were nurses, female, and worked in internal medicine/geriatrics or surgical wards. More than half of the participants reported no previous HH training. The mean total score on the WHO HH Knowledge Questionnaire was 25.63 (SD = 5.79), with observed scores ranging from 7 to 46. The internal consistency of the 46 dichotomous scoreable elements was acceptable (KR-20 = 0.79). Some baseline characteristics differed significantly between centers, including gender distribution, work setting, previous HH training, and knowledge scores.

Detailed baseline scanner measurements are reported in Table 2. At baseline, 44.2% of participants failed the scanner assessment, with dorsum areas being the most frequently insufficiently covered. Rings were detected in 36.0% of participants.

As reported in Table 3, baseline scanner pass/fail status did not differ significantly by profession or baseline HH knowledge score, whereas statistically significant differences were observed by gender and age.

Table 4 shows scanner pass rates and participant retention at each time-point, stratified by center, for a total of 2422 scanner assessments. Pass rates generally improved over time, although patterns differed across centers and participant retention declined during follow-up. Retention refers to the proportion of baseline participants from each center who completed each scanner assessment and does not indicate HH compliance in clinical practice.

Pass rates refer to the proportion of completed scanner assessments classified as passed at each time-point. Assessment labels are grouped by scheduled follow-up period; repeated assessments within the same period were performed within the same assessment round. Vertical dashed lines separate scheduled follow-up periods. Denominators for each center and time-point are reported in Table 4. Missing points indicate time-points not collected in the corresponding center.

**Figure 1:**
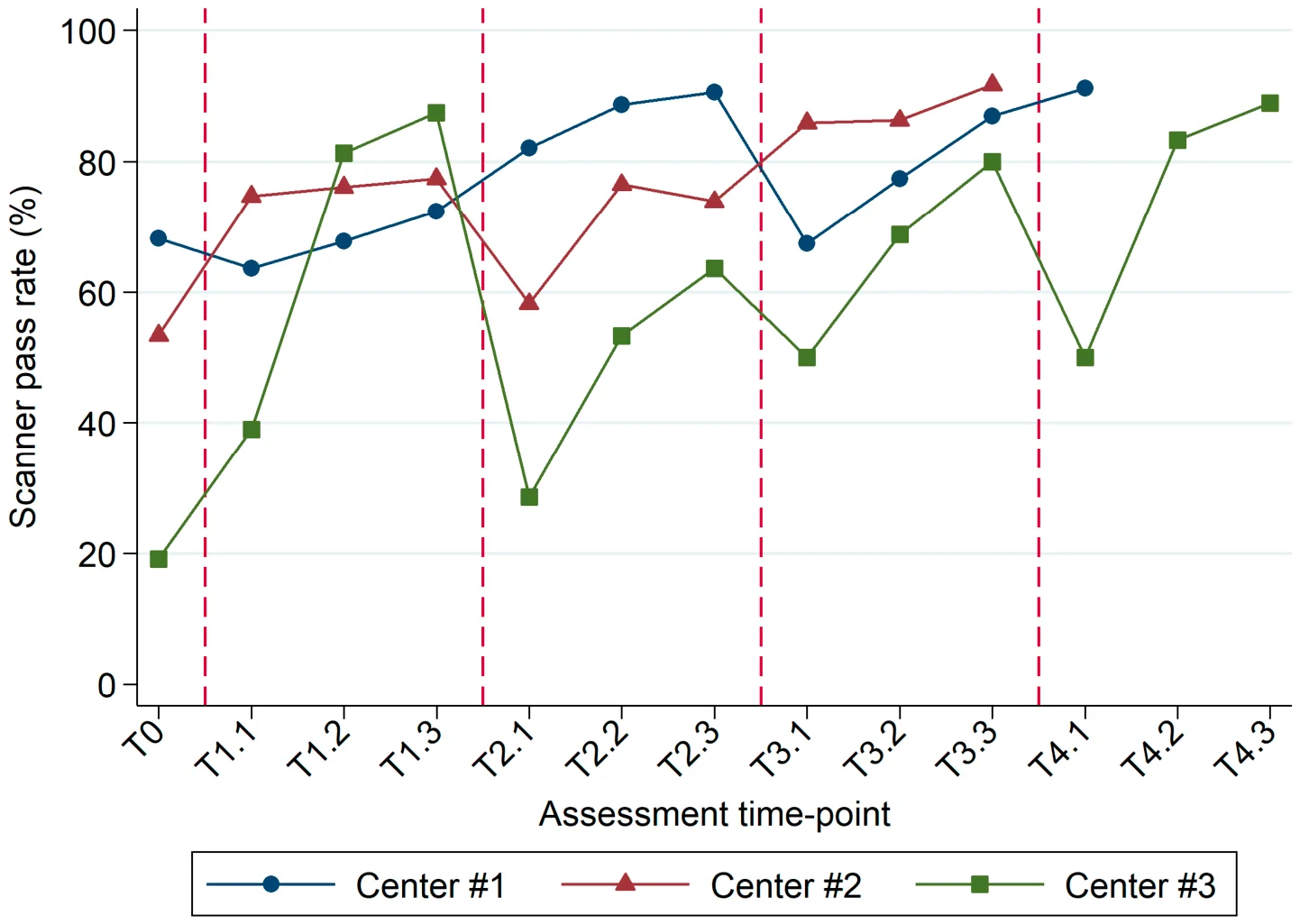
Scanner pass rates over time by center.

Figure 1 provides a visual summary of scanner pass rates over time by center. Overall, pass rates tended to increase after the baseline assessment, although patterns varied across centers and follow-up time-points. Center #1 showed a relatively progressive improvement over time, Center #2 showed higher pass rates during later follow-up assessments, and Center #3 showed greater variability, partly reflecting smaller denominators and lower participant retention at later assessments.

Retention analyses were performed to assess whether loss to follow-up was potentially selective. Overall, 237/292 participants (81.2%) completed at least one T3 assessment, whereas 164/292 participants (56.2%) completed the final common follow-up assessment at T3.3. Completion of T3.3 differed significantly by center, gender, previous HH training, and baseline scanner performance. In particular, participants who passed the baseline scanner assessment were more likely to complete T3.3 than those who failed at baseline, suggesting a potential selection effect. Individual reasons for missed follow-up assessments were not systematically recorded because participants were followed using non-identifiable study codes and no direct contact details were collected. Loss to follow-up was therefore described using retention at each scanner assessment. Missed assessments were likely related to routine organizational constraints, shift patterns, workload, rotating scanner availability, and staff turnover.

**Figure 2:**
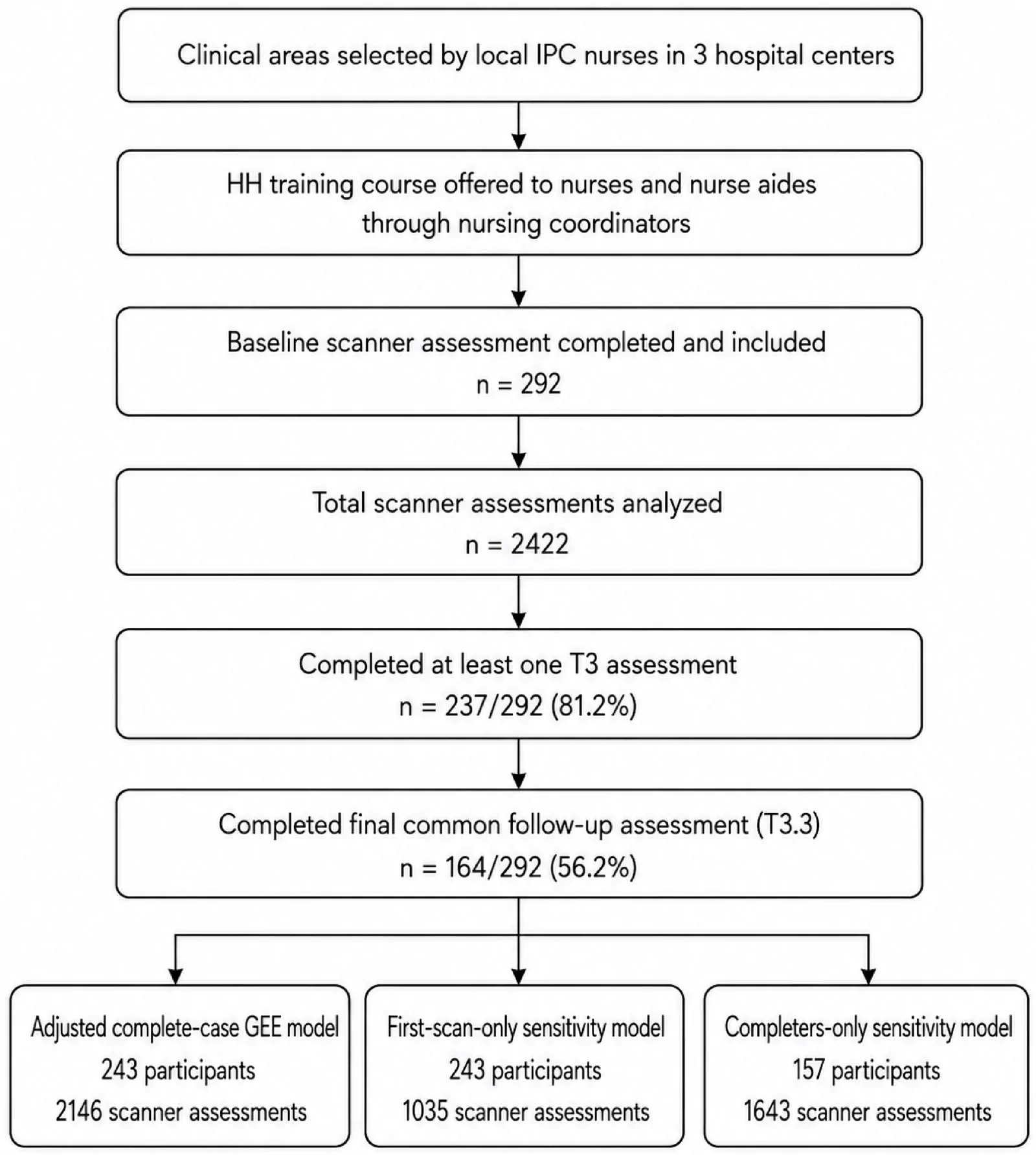
Participant flow and analysis samples. The total number of potentially eligible healthcare workers in the participating wards was not available; therefore, a recruitment rate could not be calculated. T3.3 was the final common follow-up assessment available across all three centers. The complete-case GEE models included participants with complete baseline covariate data.

Participant flow, follow-up completion, and analysis samples are summarized in Figure 2.

Figure 1 also shows a recurrent within-period pattern. Within most follow-up periods, pass rates increased across repeated assessments performed during the same assessment round, whereas the first assessment of the subsequent period was often lower than the last assessment of the previous period. For example, in Center #1, pass rates increased from 67.4% at T3.1 to 86.9% at T3.3, but decreased from 90.6% at T2.3 to 67.4% at T3.1. Similar decay-and-recovery patterns were observed in Centers #2 and #3. This pattern suggests that part of the observed improvement may reflect immediate within-session practice or feedback effects, although the first-scan-only sensitivity analysis still showed a significant temporal trend.

To formally assess the longitudinal trend in scanner performance, GEE logistic regression models with robust standard errors were fitted, accounting for repeated scanner assessments within participants. In the adjusted model including all scanner assessments with complete covariate data, each additional scheduled month from baseline was associated with higher odds of passing the scanner assessment (OR = 1.18, 95% CI 1.13–1.24, *p* < 0.001) (Table 5). When time was modeled categorically in the same adjusted framework, the overall effect of period was statistically significant (χ^2^(4) = 64.75, *p* < 0.001), with higher odds of passing at each follow-up period compared with baseline. To reduce the potential influence of within-session repeated practice, a sensitivity analysis was performed by restricting the model to the first scanner assessment of each follow-up period. The temporal trend remained statistically significant (OR = 1.16, 95% CI 1.11–1.21, *p* < 0.001), and the overall categorical effect of period also remained significant (χ^2^(4) = 35.86, *p* < 0.001). In this sensitivity analysis, T1 was not significantly different from baseline, whereas T2, T3, and T4 showed significantly higher odds of passing the scanner assessment. The completers-only sensitivity analysis included 157 participants with complete covariate data who completed the final common follow-up assessment at T3.3, corresponding to 1643 scanner assessments. In this model, each additional scheduled month from baseline remained associated with higher odds of passing the scanner assessment (OR = 1.19, 95% CI 1.13–1.26, *p* < 0.001). The same finding was confirmed in the completers-only first-scan-only analysis (OR = 1.16, 95% CI 1.09–1.22, *p* < 0.001). Across the main and first-scan-only models, ring detection was associated with lower odds of passing the scanner assessment, whereas baseline knowledge score was not independently associated with scanner performance after adjustment for center, profession, gender, age, previous hand hygiene training, and ring detection. Ring detection did not show a monotonic decrease over follow-up, being detected in 36.0% of assessments at T0, 38.6% at T1, 32.7% at T2, 36.9% at T3, and 31.7% at T4. In a sensitivity model excluding ring detection from the covariates, the association between scheduled month from baseline and scanner pass rate remained virtually unchanged (OR = 1.19, 95% CI 1.13–1.24, *p* < 0.001). This was also confirmed in the first-scan-only model without ring adjustment (OR = 1.16, 95% CI 1.11–1.22, *p* < 0.001).

## 4. Discussion

The aim of this multicenter quality improvement project was to assess scanner-measured HH performance over time after a standardized HH training program supported by an automated visual feedback scanner among nurses and nurse aides in three Italian hospital centers. Overall, scanner pass rates improved during follow-up, and time remained significantly associated with higher odds of passing the scanner assessment in adjusted GEE models. This association was also observed in the first-scan-only sensitivity analysis, suggesting that the longitudinal trend was not solely explained by immediate repeated practice within the same session. However, because of the QI design, the absence of a control group, and the selective loss to follow-up, these findings should be interpreted as an association rather than evidence of causal effectiveness. At baseline, a substantial proportion of participants failed the scanner assessment, with dorsum areas being the most frequently insufficiently covered. This finding is consistent with previous literature showing that healthcare workers may neglect specific hand areas during HH procedures [18,19]. From an educational perspective, this supports the potential usefulness of visual feedback tools: by making insufficiently covered areas immediately visible, scanner-based systems may help healthcare workers recognize and correct technical gaps that are difficult to identify through traditional frontal teaching alone.

Ring detection was consistently associated with lower odds of passing the scanner assessment. This result is relevant because hand and wrist jewelry are recognized as potential barriers to effective HH and may contribute to microbial contamination [20,21]. In this project, participants were informed about the importance of removing jewelry but were not required to remove rings before scanner assessments, in order to reflect routine practice in a real-world QI context. Therefore, the observed association between ring detection and scanner failure reinforces the importance of including jewelry removal and related behavioral barriers in HH training and feedback activities. Baseline HH knowledge was not independently associated with scanner performance in the adjusted longitudinal models. This finding does not imply that knowledge is irrelevant, but it supports the view that knowledge alone is insufficient to ensure adequate HH technique. HH behavior and technique are influenced by multiple factors, including habits, workload, time pressure, environmental constraints, local safety culture, and opportunities for feedback [6]. Therefore, scanner-supported feedback should be interpreted as a possible adjunct within a broader multimodal strategy, rather than as a stand-alone solution. Similarly, although gender and age differed by baseline scanner pass/fail status in crude analyses, these associations were attenuated or inconsistent in the adjusted longitudinal models and were therefore not interpreted as robust independent predictors of scanner performance.

A key finding was the progressive decline in participation across follow-up assessments. Retention analysis showed that loss of follow-up was not completely random: participants who performed better at baseline were more likely to complete the final common follow-up assessment. This suggests a potential selection effect, whereby more motivated or technically proficient participants may have been more likely to remain engaged over time. Consequently, the observed increase in pass rates may partly reflect selective retention, in addition to potential improvements related to training and scanner-based feedback. This issue is particularly important in QI projects conducted in real clinical settings, where workload, staff turnover, and organizational constraints can affect participation over time. In the participating centers, high staff turnover during the observation period, partly related to an open public recruitment competition, may have further contributed to declining retention.

Differences between centers were also observed. These differences may reflect variation in baseline HH technique, local organization, scanner availability, training schedules, staffing dynamics, or institutional IPC maturity. A formal assessment of IPC maturity, such as IPCAF, was not performed before implementation; therefore, the extent to which center-level organizational characteristics influenced the results cannot be fully assessed. Future studies should consider collecting center-level implementation data to better understand how local context affects the uptake and sustainability of scanner-supported HH training.

The dual role of the scanner should also be considered. In this project, the scanner functioned both as an educational feedback tool and as the outcome measure. This creates an inherent risk of task-specific learning or increased familiarity with the device. The observed decay-and-recovery pattern across repeated assessments further supports this cautious interpretation. Improvements within the same assessment round may reflect immediate practice or feedback effects, whereas decreases at the first assessment of subsequent periods suggest that part of the gain may not have been fully retained between follow-up periods. The first-scan-only sensitivity analysis was conducted to reduce the influence of immediate within-session practice and showed that the temporal trend remained significant. Nevertheless, this analysis cannot fully distinguish sustained improvement in HH technique from familiarity with the assessment procedure. Controlled studies using independent outcome measures are needed to clarify this issue. Importantly, the outcome of this project was scanner-measured hand-rubbing technique under test conditions, not real-world HH compliance in clinical care. The scanner can objectively assess whether the hand surface is adequately covered during a simulated alcohol-based hand rub procedure, but it does not measure whether healthcare workers perform HH at the appropriate moments of care, as defined by the WHO “Five Moments for Hand Hygiene” framework [20]. Therefore, the findings should not be interpreted as evidence of improved clinical HH compliance or reduced HAIs. Future studies should combine scanner-based technique assessment with direct observation, electronic monitoring, or other measures of real-world HH behavior.

Several limitations must be acknowledged. First, the QI design and absence of a control group limit causal inference. Second, participating centers were selected by convenience based on access to the scanner, and the total number of potentially eligible healthcare workers in the participating wards was not available; therefore, a recruitment rate could not be calculated. Third, follow-up assessments were not uniform across centers, with T4 available only in two centers, and participant retention declined over time. Relatedly, standard unweighted GEE models rely on a non-informative missingness assumption for outcome data. Although the completers-only sensitivity analysis yielded results consistent with the main analysis, selective retention cannot be fully excluded. Adjusted models were also based on complete covariate data; participants included in the complete-case models differed from excluded participants by center, gender, and baseline scanner performance, suggesting that complete-case estimates should also be interpreted cautiously. Fourth, the sample included only nurses and nurse aides, limiting generalizability to other healthcare professionals. Fifth, no formal assessment of institutional IPC maturity was performed. Residual hospital-level clustering and unmeasured center-level contextual effects cannot be excluded. Sixth, although scanner-based assessment provides an objective evaluation of hand-rubbing coverage, participants were aware that their technique was being assessed; therefore, test-specific behavior or a Hawthorne-like effect cannot be excluded [22]. Finally, the scanner was both the feedback tool and the outcome measure, and the study did not assess real-world HH compliance, acceptability, workflow integration, or patient-level outcomes.

Despite these limitations, this project provides pragmatic multicenter QI data on scanner-supported HH training in Italian hospital settings. The findings suggest that immediate visual feedback may help support improvement in scanner-measured HH technique, especially when integrated into standardized training delivered by IPC nurses. However, implementation should be embedded within broader multimodal HH strategies, and future controlled studies should assess sustainability, feasibility, user acceptability, and impact on real-world HH compliance.

## 5. Conclusions

This multicenter quality improvement project suggests that a standardized HH training program supported by scanner-based immediate feedback was associated with improved scanner-measured HH technique over time among nurses and nurse aides. Ring detection was consistently associated with lower odds of passing the scanner assessment, highlighting the importance of addressing hand and wrist jewelry in HH education. However, the absence of a control group, the dual role of the scanner as both feedback tool and outcome measure, and selective retention across follow-up assessments limit causal interpretation. Scanner-based feedback may represent a useful adjunct to traditional HH education within broader multimodal strategies, but further controlled studies are needed to assess sustainability, implementation feasibility, user acceptability, and impact on real-world HH compliance.

## Figures and Tables

**Table 1:** Characteristics of the participants (*n* = 292).

	Total	Center #1 (*n* = 157)	Center #2 (*n* = 88)	Center #3 (*n* = 47)	*p*-Value
Job title (obs = 292), *n* (%) Nurse Nurse aides	202 (69.2) 90 (30.8)	100 (63.7) 57 (36.3)	64 (72.7) 24 (27.3)	38 (80.9) 9 (19.1)	0.057
Gender (obs = 264), *n* (%) Female Male	214 (81.1) 50 (18.9)	138 (88.5) 18 (11.5)	53 (74.6) 18 (25.4)	23 (62.2) 14 (37.8)	<0.001
Age (obs = 244), mean (SD)	41.99 (10.98)	42.07 (10.78)	41.79 (11.49)	42.05 (11.15)	0.984
Setting (obs = 292), *n* (%) Internal Medicine or Geriatrics Surgery ICU	132 (45.2) 106 (36.3) 54 (18.5)	92 (58.6) 65 (41.4) --	23 (26.1) 29 (33.0) 36 (40.9)	17 (36.2) 12 (25.5) 18 (38.3)	<0.001
Previous training courses on HH (obs = 266), *n* (%) Yes No	112 (42.1) 154 (57.9)	31 (19.8) 126 (80.2)	58 (80.6) 14 (19.4)	23 (62.2) 14 (37.8)	<0.001
WHO HH Knowledge Questionnaire for Health Care Workers total score (obs = 266), mean (SD)	25.63 (5.79)	24.78 (4.92)	26.84 (7.53)	26.70 (4.70)	0.019

Legend: obs = number of observations; SD = standard deviation; ICU = Intensive Care Unit; HH = hand hygiene.

**Table 2:** Baseline HH performance assessed by scanner (*n* = 292).

	Number of Participants (%)
Left palm,	
Fail	66 (22.6)
Pass	226 (77.4)
Right palm,	
Fail	40 (13.7)
Pass	252 (86.3)
Left dorsum,	
Fail	78 (26.7)
Pass	214 (73.3)
Right dorsum,	
Fail	74 (25.3)
Pass	218 (74.7)
Total evaluation,	
Fail	129 (44.2)
Pass	163 (55.8)
Ring detected (Yes)	105 (36.0)

**Table 3:** Differences between groups by baseline scanner pass/fail status.

	Failed	Passed	*p*-Value
Job title (obs = 292), *n*(%) Nurse Nurse Aides	87 (43.1) 42 (46.7)	115 (56.9) 48 (53.3)	0.568
Gender (obs = 264), *n*(%) Female Male	79 (36.9) 34 (68.0)	135 (63.1) 16 (32.0)	<0.001
Age (obs = 244), mean (SD)	44.17 (11.11)	40.56 (10.58)	0.011
HH knowledge score (baseline; obs = 266), mean (SD); [0 ≤ score ≤ 46]	24.96 (6.89)	26.14 (4.75)	0.099

Legend: SD = standard deviation; HH = hand hygiene.

**Table 4:** Scanner pass rates and participant retention by center and time-point.

	Center #1			Center #2			Center #3		
Time-Point	N° of Participants	Test Passed (%)	Retention (%)	N° of Participants	Test Passed (%)	Retention (%)	N° of Participants	Test Passed (%)	Retention (%)
T0	157	68.2	100	88	53.4	100	47	19.1	100
T1.1	154	63.6	98	75	74.7	85	41	39.0	87
T1.2	152	67.8	97	67	76.1	76	32	81.3	68
T1.3	145	72.4	92	53	77.4	60	24	87.5	51
T2.1	145	82.1	92	67	58.2	76	21	28.6	45
T2.2	142	88.7	90	51	76.5	58	15	53.3	32
T2.3	128	90.6	82	23	73.9	26	11	63.6	23
T3.1	141	67.4	90	71	85.9	81	24	50.0	51
T3.2	137	77.4	87	51	86.3	58	16	68.8	34
T3.3	130	86.9	83	24	91.7	27	10	80.0	21
T4.1	137	91.2	87	N/C	N/C	N/C	22	50.0	47
T4.2	N/C	N/C	N/C	N/C	N/C	N/C	12	83.3	26
T4.3	N/C	N/C	N/C	N/C	N/C	N/C	9	88.9	19

Legend: N/C = not collected. Retention (%) refers to the proportion of baseline participants from each center who completed the scanner assessment at each time-point. It does not indicate hand hygiene compliance in clinical practice.

**Table 5:** Adjusted generalized estimating equation models of scanner pass rates over time.

Predictor	Model 1: All Scans OR [95% CI]	*p*-Value	Model 2: First Scans Only OR [95% CI]	*p*-Value
Time, per scheduled month	1.185 [1.131–1.241]	<0.001	1.158 [1.105–1.214]	<0.001
Center #2 vs. Center #1	0.719 [0.488–1.059]	0.095	0.785 [0.481–1.280]	0.331
Center #3 vs. Center #1	0.277 [0.173–0.442]	<0.001	0.202 [0.111–0.369]	<0.001
Nurse vs. nurse aide	1.169 [0.892–1.532]	0.257	1.170 [0.816–1.676]	0.393
Female vs. male	1.279 [0.958–1.707]	0.095	1.401 [0.949–2.070]	0.090
Age, per year	0.983 [0.971–0.995]	0.008	0.987 [0.973–1.002]	0.098
Previous HH training	1.329 [0.986–1.790]	0.062	1.213 [0.810–1.816]	0.349
Baseline knowledge score	1.018 [0.996–1.040]	0.106	1.022 [0.999–1.045]	0.065
Ring detected	0.632 [0.495–0.807]	<0.001	0.692 [0.512–0.934]	0.016

Legend: OR = odds ratio; CI = confidence interval; HH = hand hygiene. Odds ratios were estimated using GEE logistic regression models with robust standard errors clustered by participant ID and an exchangeable working correlation structure. Model 1 included all scanner assessments with complete covariate data, corresponding to 2146 scans from 243 participants. Model 2 was restricted to the first scanner assessment of each follow-up period to reduce the influence of within-session repeated practice, corresponding to 1035 scans from 243 participants. Both models were adjusted for center, profession, gender, age, previous hand hygiene training, baseline knowledge score, and ring detection. Analyses were conducted as complete-case analyses because of missing baseline covariates.

## Data Availability

The data presented in this study are not publicly available due to institutional and privacy considerations related to the quality improvement nature of the project. Aggregated data may be made available from the corresponding author upon reasonable request.

## Public Involvement Statement

No public involvement in any aspect of this research.

## Guidelines and Standards Statement

This manuscript was drafted against the SQUIRE reporting guideline for Quality Improvement studies.

## Abbreviations

The following abbreviations are used in this manuscript:

GEE	Generalized estimating equations
HAIs	Healthcare-associated infections
HH	Hand hygiene
ICU	Intensive care unit
IPC	Infection prevention and control
QI	Quality improvement
WHO	World Health Organization
